# Skeletal Muscle Changes After Elective Colorectal Cancer Resection: A Longitudinal Study

**DOI:** 10.1245/s10434-016-5188-1

**Published:** 2016-03-22

**Authors:** George Malietzis, Andrew C. Currie, Neil Johns, Kenneth C. Fearon, Ara Darzi, Robin H. Kennedy, Thanos Athanasiou, John T. Jenkins

**Affiliations:** Department of Surgery, St Marks Hospital, Middlesex, UK; Department of Surgery and Cancer, Imperial College, London, UK; Department of Clinical and Surgical Sciences, University of Edinburgh, Edinburgh, UK

## Abstract

**Background:**

Muscle depletion is a poor prognostic indicator in colorectal cancer (CRC) patients, but there were no data assessing comparative temporal body composition changes following elective CRC surgery. We examined patient skeletal muscle index trajectories over time after surgery and determined factors that may contribute to those alterations.

**Methods:**

Patients diagnosed with CRC undergoing elective surgical resection between 2006 and 2013 were included in this study. Image analysis of serial computed tomography (CT) scans was used to calculate lumbar skeletal muscle index (LSMI). A multilevel mixed-effect linear regression model was applied using STATA (version 12.0) using the *xtmixed* command to fit growth curve models (GCM) for LSMI and time.

**Results:**

In 856 patients, a total of 2136 CT images were analyzed; 856 (38.2 %) were preoperative. A quadratic GCM with random intercept and random slope for patients’ LSMI was identified that demonstrated laparoscopy produces a positive change on the LSMI curve [estimate = 0.17 cm^2^/m^2^, standard error (SE) 0.06 cm^2^/m^2^; *p* = 0.03], whereas Union for International Cancer Control (UICC) stage III + IV disease contributed to a negative curve change (estimate = −0.19 cm^2^/m^2^, SE 0.09 cm^2^/m^2^; *p* = 0.03). Older age (*p* < 0.01), female gender (*p* < 0.01), higher American Society of Anesthesiologists (ASA) score (*p* < 0.01), and altered systemic inflammatory response [SIR] (*p* = 0.03) were factors significantly associated with lower values of LSMI over time.

**Conclusion:**

In patients undergoing CRC surgery, laparoscopy and the absence of a significantly elevated SIR favored preservation and restoration of skeletal muscle, postoperatively. These emerging data may permit the development of new treatment protocols whereby monitoring and modification of body composition has therapeutic potential.

It is becoming increasingly clear that a variety of body composition changes occur in cancer patients, and that muscle depletion is a common, albeit in most, occult feature. Muscle depletion is characterized by reduction in muscle size (myopenia) and an increased infiltration by inter- and intramuscular fat, described as myosteatosis.^1^,^2^ The incidence of muscle depletion varies from 15 to 70 % for patients treated for CRC,^3^ and evidence also demonstrates that muscle depletion is associated with poorer outcomes in patients treated for cancer.^4^,^5^ For CRC patients treated surgically, myopenia negatively impacts short-term outcomes, including mortality, morbidity, and functional recovery.^3^ Muscle depletion, estimated from CTBC analysis, has also been found to be a prognostic factor for developing severe toxicity in CRC patients receiving chemotherapy.^6^ Finally, emerging data suggest that myopenia can be an independent predictor of poorer survival after CRC treatment; however, neither the point of onset of muscle depletion nor the patterns of muscle alterations over time and their precipitants are known. Therefore, there is a need to identify whether body composition changes assessable by surveillance imaging after CRC can be related to specific clinicopathological or treatment factors that, once identified, might allow muscle depletion to be contained or modified.

Growth curve modeling (GCM) is an advanced method for demonstrating within-patient and between-patient variation in outcomes measured across different time points over a follow-up period.^7^ The GCM approach has allowed researchers to overcome problems in studies that assess comparisons of the intraindividual changes over time. More traditional methods examining changes of time, such as analysis of variance and analysis of covariance, are problematic and are limited, mandating accuracy in equal group sizes, a condition that is very difficult to meet.^8^,^9^

Using the GCM approach, we aimed to not only examine how patient body composition, as determined by skeletal muscle index trajectories, varied over time after elective surgery for CRC but also to determine specific factors that may contribute to alterations over time.

## Methods

### Patient Population

Overall, 1477 consecutive patients undergoing CRC surgery at St Mark’s Hospital, London, between January 2006 and December 2013, were identified from a prospective database. Patients with recorded height data, laboratory blood test data within 4 weeks of staging computed tomography (CT) scan and preoperative staging, and surveillance CT images stored and retrievable in an electronic format suitable for image analysis were included in the study. Exclusions were patients with disease recurrence confirmed preoperatively or at surgery, and emergency operations. All prospectively recorded clinical and pathological data were revalidated from medical and histopathology records. Data collected prospectively during the perioperative period (within 30 days of surgery) included age, sex, body mass index (BMI), American Society of Anesthesiologists (ASA) physical status classification system, tumor site, TNM stage [Union for International Cancer Control (UICC) 5 version] and surgical approach. Laboratory blood test data collected included preoperative neutrophil and lymphocyte counts. The neutrophil to lymphocyte ratio (NLR) was derived as a valid reflection of the host systemic inflammatory response (SIR);^10^ a high NLR [HNLR] was defined as >3.0.

### Body Composition Analysis

Serial CT scan images performed as part of the CRC follow-up protocol were retrieved from digital storage in the picture archiving and communication system (PACS). CT image analysis *Slice*-*O*-*Matic* V4.3 software (Tomovision, Montreal, QC, Canada) was performed as described previously.^11^ Briefly, total skeletal muscle surface areas (cm^2^) were evaluated on a single image at the third lumbar vertebrae (L3) using Hounsfield unit (HU) thresholds of −29 to 150 for skeletal muscle, −50 to 150 for visceral adipose tissue, and −190 to −30 for subcutaneous adipose tissues. The sum of skeletal cross-sectional muscle areas was normalized for stature (m^2^) and reported as lumbar skeletal muscle index (LSMI) [cm^2^m^−2^].

### Data Analysis

A non-parametric Mann–Whitney *U* test was used to determine significant differences between the LSMI from baseline demographic and clinicopathological characteristics (a *p* value < 0.05 was regarded as significant), and a multi-level, mixed-effect GCM was applied using STATA version 12.0 (StataCorp LP, College Station, TX, USA).^12^ GCM is a special case of random–coefficient models where the coefficient of time varies randomly between subjects. Growth trajectories can take a variety of shapes. A flexible approach to model possible non-linear growth in *Y*_ij_ is to use a pth degree polynomial function of time ${t}_{\text{ij}} ,_{{}} {Y}_{{{\text{ij}} }} = {\text{b1 }}\left( {\text{constant}} \right) \, + {\text{b2}}.{t}_{\text{ij}} \left( {\text{linear}} \right)_{{}} + {\text{ b3}}.{t}^{ 2}_{\text{ij}} \left( {\text{quadratic}} \right) \, + \ldots + {\text{ b}}.{\text{p }} + { 1t}^{\text{p}}_{\text{ij}} + {\varvec{\upxi}}_{\text{ij}}$.

Using the *xtmixed* command, we modeled the shape of trajectories of the dependent variable (LSMI) over time and how these trajectories varied due to time- and patient-level covariates. A number of steps were considered in specifying a repeated measures analysis using a GCM approach, as described by Rabe-Hesketh and Skrondal.^12^ Penalized-likelihood information criteria, such as Akaike’s information criterion (AIC) and the Bayesian information criterion (BIC) were used for model selection. Heteroscedasticity checks were also performed. Heteroscedasticity refers to the circumstance in which the observed variance is independent of the variable mean.^13^

## Results

### Study Population

The clinicopathological characteristics of the 856 elective colorectal cancer (CRC) resection cases that fulfilled the selection criteria are provided in Table 1. The median age at operation was 67 years [interquartile range (IQR) 58–76]. Overall, 63.1 % of patients were treated laparoscopically [intention-to-treat] (63.1 % laparoscopic vs. 36.9 % open); 238 (27.8 %) were rectal cancers. The majority of the operations were performed or supervised by one of two consultant colorectal surgeons (RHK and JTJ) who were designated CRC surgeons and were both trained in laparoscopic colorectal surgery, with the laparoscopic technique being standardized between surgeons. These two surgeons performed both open and laparoscopic colorectal resections. Additional cases were performed by three other surgeons using mainly open techniques or by laparoscopy, with mentoring by the two laparoscopic colorectal surgeons. The selection for open or laparoscopic surgery reflected each individual surgeon’s expertise during the study period, and all patients were part of an enhanced recovery after surgery (ERAS) protocol for recovery. A total of 2136 CT images were analyzed, of which 856 (38.2 %) were pretreatment scans.

**Table 1:** Clinicopathological characteristics of the 856 elective colorectal cancer resection cases that fulfilled the selection criteria

Baseline demographics (*N* = 856)	Count	%
Gender		
Male	482	56.3
Female	374	43.7
Age category (years)		
<65	389	45.4
≥65	467	54.6
NLR category		
Low	318	37.1
High	268	62.9
ASA status		
1 + 2	711	83.1
3 + 4	145	16.9
Surgical approach		
Open	316	36.9
Laparoscopic	540	63.1
Tumor site		
Colon	618	72.2
Rectum	238	27.8
UICC stage		
I	197	23.0
II	302	35.3
III	283	33.1
IV	74	8.6
BMI categories		
Underweight BMI < 18.5	18	2.2
Normal BMI (18.5–25)	276	32.1
Overweight BMI (25–30)	349	40.8
Obese BMI (>30)	213	24.9

*NLR* neutrophil to lymphocyte ratio, *ASA* American Society of Anesthesiologists physical status, *UICC* Union for International Cancer Control, *BMI* body mass index

### Body Composition Analysis

The median LSMI was 42.9 (IQR 37.4–49.5) cm^2^m^2^, and men had a higher LSMI compared with women (*p* < 0.001). Elderly patients, patients with a high ASA or high NLR preoperatively, or patients with a colon cancer had significantly lower LSMI median values compared with patients aged <65 years (*p* < 0.001), ASA I + II (*p* < 0.001), NLR < 3.0 (*p* < 0.001), and patients with rectal cancers (*p* = 0.016), respectively. No differences were noted between the preoperative LSMI and the type of surgical approach (laparoscopic vs. open; *p* = 0.710) or the UICC stage (*p* = 0.056). The CT-derived LSMI values and their associations with different clinicopathological variables are summarized in Table 2.

**Table 2:** LSMI values calculated from the CT analysis and their relationships with the clinicopathological variables

	L3 muscle index (LSMI)			
	Median	25th percentile	75th percentile	*p* value
Gender				
Male	47.16	41.20	54.09	**<0.001**
Female	39.20	34.80	43.56	
Age category (years)				
<65	45.42	39.58	51.80	**<0.001**
≥65	41.55	35.91	47.66	
NLR category				
Low	44.25	37.57	52.62	**<0.001**
High	41.56	36.36	47.47	
ASA status				
I + II	44.64	38.90	51.13	**<0.001**
III + IV	40.52	35.72	45.56	
Surgical approach				
Open	43.12	36.31	51.18	0.710
Laparoscopic	43.79	37.90	49.68	
Tumor site				
Colon	42.81	37.46	49.38	**0.016**
Rectum	44.73	38.20	51.76	
UICC stage				
I	45.26	37.69	52.53	0.056
II	41.90	36.86	49.38	
III	44.36	38.52	50.35	
IV	42.66	37.91	46.88	

*NLR* neutrophil to lymphocyte ratio, *ASA* American Society of Anesthesiologists, *UICC* Union for International Cancer Control, *L3* third lumbar vertebrae, *LSMI* lumbar skeletal muscle index, *CT* computed tomography

Bold *p* values indicate statistical significance at *p* < 0.05

### Model Fit Non-linear Growth

Patient LSMI change was non-linear over time when the observed growth trajectories were plotted. As the relationship between LSMI over time was non-linear, we included a quadratic term for time in our model; both time terms in this model were statistically significant (if the time^2^ term had not been statistically significant, we could have only included a linear term for time in our model). The estimated standard deviation (SD) of the random intercept was 8.53 (95 % confidence interval [CI] 8.09–9.00) and the estimated SD of the error was 3.22 (95 % CI 3.09–3.36). We then included a random slope on time, to permit variability between patients in relation to overall rates of LSMI change. The SD of the random coefficient on time was 0.27 (95 % CI 0.14–0.53), indicating heterogeneity between the rates of change in LSMI. In addition, the estimated SD of the error term decreased from 3.22 to 3.17, indicating a better fit of the model.

### Quadratic Growth for Patients’ LSMI that Includes Patient-Level Covariates

#### Gender

At any given time, we estimated that a female patient’s LSMI was 7.95 cm^2^/m^2^ [standard error (SE) 0.58 cm^2^/m^2^] less than a male patient’s LSMI. The coefficients of both time and time^2^ were significant at the 5 % level. Gender did not exert any effect on the slope of the curve trajectory as the estimate for gender*time variable was not significant from the two-stage model formation.

#### Age

Elderly patients (>65 years of age) had an LSMI, on average, of 3.79 cm^2^/m^2^ (SE 0.11 cm^2^/m^2^) less than younger patients (< 65years of age). Inclusion of the age*time variable in a two-stage formation model was not significant and the fact that the AIC and BIC values were in favor of the polynomial model; the two-stage formation model was omitted.

#### Preoperative Systemic Inflammatory Response

Elevated preoperative SIR, expressed as NLR > 3.0 (HNLR), had a significant effect on the LSMI trajectory over time. Patients with HNLR at any given time point had a lower LSMI at an average of −2.28 cm^2^/m^2^ (SE 0.79 cm^2^/m^2^) compared with the patients with an NLR < 3.0 (low NLR). The two-stage formation model had the best-fit values, but NLR did not have an impact on the slope of the trajectory.

#### American Society of Anesthesiologists

The two-stage formation model was also the best-fit model when ASA was considered as a patient-level covariate. Patients with a higher ASA score (III + IV) had a significantly lower LSMI (estimate = −2.68 cm^2^/m^2^; SE 0.62 cm^2^/m^2^) than patients with an ASA score of I or II.

#### Surgical Approach

At any given time, for patients who underwent laparoscopic resection we estimated that their LSMI was not statistically different than the LSMI of patients who had an open procedure. The coefficients of time^2^ were significant at the 5 % level. The surgical approach had an impact on the slope of LSMI trajectory as the estimate for surgical approach*time variable was significant from the two-stage model formation. Of interest, patients who underwent a laparoscopic resection had a positive change to the slope of their LSMI trajectory compared with the open approach group (estimate = +0.15 cm^2^/m^2^; SE 0.06 cm^2^/m^2^). No significant differences were observed between the preoperative BMI (*p* = 0.61), ASA (*p* = 0.09), age (*p* = 0.21), NLR (*p* = 0.91), and the type of surgical approach.

#### Tumor Site

The two-stage formation model was the best-fit model when tumor location was considered as the patient-level covariate. Patients who underwent surgery for a rectal tumor had a negative change to the slope of their LSMI trajectory compared with the colon group (estimate = −0.13 cm^2^/m^2^; SE 0.05 cm^2^/m^2^).

#### Union for International Cancer Control (UICC) Stage

Patients with a higher stage of disease (UICC stage III + IV) had a negative change to the slope of their LSMI trajectory compared with patients with UICC stage I + II (estimate = −0.20 cm^2^/m^2^; SE 0.09 cm^2^/m^2^).

#### Heteroscedasticity

Heteroscedasticity checks were performed for each of the patient-level covariates under investigation, and no evidence of heteroscedasticity was identified.

**Fig. 1:**
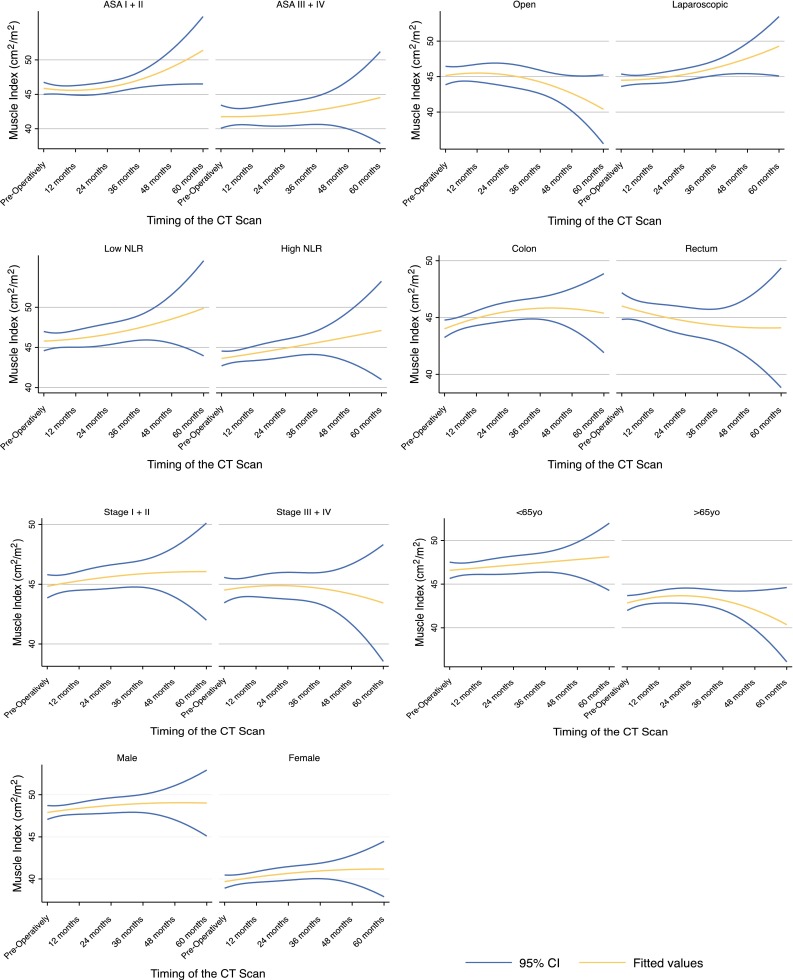
Mean trajectory and 95 % range of patient-specific trajectories of the LSMI for the different patient-level covariates from the quadratic model. *LMSI* lumbar skeletal muscle index, *ASA* American Society of Anesthesiologists, *CT* computed tomography, *NLR* neutrophil to lymphocyte ratio, *yo* years old

Figure 1 shows the mean trajectory and 95 % range of patient-specific trajectories for the different patient-level covariates. Table 3 summarizes the maximum likelihood estimates for quadratic models for the patients’ LSMI.

**Table 3:** Maximum likelihood estimates for quadratic models when the different patient-level factors were investigated

Skeletal muscle index	Gender (female vs. male)		Age, years (>65 vs. <65)		NLR (HNLR vs. LNLR)		ASA (III + IV vs. I + II)		Surgical approach (laparoscopic vs. open)		Tumor site (rectum vs. colon)		UICC stage (III + IV vs. I + II)	
Best-fit model	Two-stage formation		Polynomial		Two-stage formation		Two-stage formation		Two-stage formation		Two-stage formation		Two-stage formation	
Fixed part	Estimate	SE	Estimate	SE	Estimate	SE	Estimate	SE	Estimate	SE	Estimate	SE	Estimate	SE
b 1 (constant)	47.69	(0.38)*	50.15	(0.99)*	45.68	(0.58)*	49.48	(1.65)*	44.54	(0.62)*	42.17	(0.95)*	44.21	(1.10)*
b (variable of interest)	−7.95	(0.58)*	−3.79	(0.11)*	−2.28	(0.79)*	−2.68	(0.62)*	−0.32	(0.77)	1.67	(0.09)	0.09	(0.73)
b 2 (time linear)	0.30	(0.11)*	0.33	(0.11)*	0.30	(0.13)*	0.05	(0.19)	0.21	(0.15)	0.52	(0.16)*	0.64	(0.17)*
b 3 (time quadratic)	−0.03	(0.01)*	−0.03	(0.01)*	−0.03	(0.01)*	−0.01	(0.02)	−0.02	(0.01)*	−0.03	(0.01)*	−0.03	(0.15)*
c (variable*time)	0.13	(0.09)			0.15	(0.10)	0.08	(0.08)	0.15	(0.06)^a^	−0.13	(0.05)*	−0.20	(0.09)*
Random part														
Bp	7.51	(0.22)	8.27	(0.09)	8.82	(0.29)	8.17	(0.29)	8.79	(0.27)	8.44	(0.24)	8.77	(0.27)
${\text{t}}^{\text{p}}_{\text{ij}}$	0.26	(0.10)	0.27	(0.10)	0.31	(0.09)	0.19	(0.16)	0.29	(0.09)	0.26	(0.09)	0.29	(0.09)
$\xi_{\text{ij}} \left( {\text{constant}} \right)$	0.24	(0.23)	0.01	(0.18)	0.05	(0.17)	0.07	(0.29)	0.09	(0.18)	0.11	(0.19)	0.05	(0.16)
Residual	3.17	(0.08)	3.17	(0.07)	2.93	(0.08)	3.14	(0.09)	3.07	(0.08)	8.44	(0.24)	3.08	(0.08)
Log likelihood	−5983.02		−6045.44		−4123.82		−4332.86		−5055.37		−6064.41		−5148.82	
AIC	11,984.02		12,106.88		8265.64		8683.71		10,118.38		12,146.82		10,315.64	
BIC	12,034.09		12,151.38		8312.36		8730.95		10,166.87		12,196.89		10,364.31	
Heteroscedasticity	Absent		Absent		Absent		Absent		Absent		Absent		Absent	

*NLR* neutrophil to lymphocyte ratio, *ASA* American Society of Anesthesiologists, *UICC* Union for International Cancer Control, *HNLR* high NLR, *LNLR* low NLR, *SE* standard error, *AIC* Akaike Information Criterion, *BIC* Bayesian Information Criterion

* *p* < 0.05

### Multivariate Quadratic Growth with Random Intercept and Random Slope for Patients’ LSMI

To adjust for all patient-level covariates and all statistically significant time covariates, interactions were considered for the formulation of the final model. Older age, female gender, high preoperative SIR (HNLR) and higher ASA were significantly associated with lower LSMI values over time. Laparoscopy and a more advanced UICC stage had a significant effect on the slope of the LSMI trajectory. Laparoscopy offered a positive change on the LSMI slope (estimate = 0.17 cm^2^/m^2^; SE 0.06 cm^2^/m^2^), whereas UICC stage III + IV contributed to a negative slope change (estimate = −0.19 cm^2^/m^2^; SE 0.09 cm^2^/m^2^). The coefficients of time and time^2^ were significant at the 5 % level. The log-likelihood and values for AIC and BIC were also improved, suggestive of a model of better fit. Table 4 shows the estimates of the multivariate quadratic growth with random intercept and random slope for patients’ LSMI.

**Table 4:** Multivariate quadratic growth with random intercept and random slope for patients LSMI

Skeletal muscle index	Estimate	(SE)	*p* value
Fixed part			
Gender			
Female versus male	−8.82	(0.88)	**<0.01**
Age (years)			
>65 versus <65	−3.52	(0.88)	**<0.01**
NLR			
HNLR versus LNLR	−1.89	(0.85)	**0.03**
ASA			
III + IV versus I + II	−3.53	1.16	**<0.01**
Surgical approach			
Laparoscopic versus Open	−0.68	1.20	0.60
Tumor site			
Rectum versus colon	−0.91	0.89	0.31
UICC stage			
III + IV versus I + II	0.27	0.86	0.75
Time*Laparoscopy	0.17	0.06	**0.03**
Time*Rectum	−0.13	0.11	0.25
Time*Stage III + IV	−0.19	0.09	**0.04**
Time linear	0.75	0.30	**0.01**
Time quadratic	−0.03	0.01	**0.02**
Random part			
Bp	7.41	0.32	
${\text{t}}^{\text{p}}_{\text{ij}}$	0.29	0.11	
$\xi_{\text{ij}} \left( {\text{constant}} \right)$	0.13	0.21	
Residual	2.45	0.07	
Log likelihood	−3038.85		
AIC	6111.71		
BIC	6195.49		

*NLR* neutrophil to lymphocyte ratio, *ASA* American Society of Anesthesiologists, *UICC* Union for International Cancer Control, *HNLR* high NLR, *LNLR* low NLR, *SE* standard error, *AIC* Akaike Information Criterion, *BIC* Bayesian Information Criterion

Bolded *p* values indicate statistical significance at *p* < 0.05

## Discussion

This study applied a flexible method for modeling the non-linear and asymmetric relationships between body composition and time for patients treated surgically for CRC, specifically addressing muscle mass as represented by LSMI. Using the multilevel GCM approach, we found that the LSMI–time relation followed a quadratic trajectory over the postoperative follow-up period of up to 60 months. We identified that patients with older age, female gender, high preoperative SIR (reported as HNLR and high ASA) have, on average, a low LSMI over time compared with their opposite groups. We also demonstrated that laparoscopy offered a positive change to the LSMI over time, whereas UICC stage III + IV was associated with a negative change, inferring that muscle mass is augmented after laparoscopy and depleted with higher cancer stages. Loss of skeletal mass due to aging is a well-recognized process, but muscle depletion can also be a consequence of chronic diseases such as cancer.^14^ The presence of comorbidities and the effect of the sex hormones are recognized factors that contribute to muscle metabolism.^15^,^16^ Richards et al. previously demonstrated that CT-derived body composition parameters vary between the two genders, but subgroup analysis of this cohort of patients did not reveal any different LSMI trajectories compared with the whole of the cohort;^17^ however, the pattern of muscle changes after treatment for cancer has not, to our knowledge, been examined in this way before.

We have previously demonstrated that, in patients undergoing CRC surgery, a low NLR favors maintenance of muscle mass postoperatively. Recent advancements in the investigation of the pathophysiology of skeletal muscle depletion and cachexia in cancer patients have suggested that inflammation could be considered the common link.^18^ Inflammation plays a vital role in the metabolic and body composition changes in cancer though five key domains: systemic inflammation, central energy balance, control of muscle metabolism/function, control of adipose tissue metabolism/function, and regulation of appetite.^19^ An ongoing state of low-grade inflammation that involves stimulation of various acute-phase proteins such as C-reactive protein and proinflammatory cytokines that enhance autophagy in skeletal muscle and inhibit the synthesis of myofibrillar proteins, may be the background mechanism of the effect of systemic inflammation on the muscle trajectory postoperatively.^19^ Our findings further support the assertion that resolution of the SIR is a potential approach to develop more effective therapies against muscle depletion and cancer cachexia.

We identified that laparoscopic resection has a positive impact on restoration of patient muscle mass postoperatively. Multiple randomized trials have confirmed that laparoscopy for CRC produces equivalent oncological outcomes as open surgery, and also produces benefits from decreased complications and hospital stay, decreased postoperative narcotic analgesia use, a faster return of bowel function, and improved cosmesis.^20^,^21^ We have now identified that additional benefits include maintenance and restoration of muscle mass. The associated tissue injuries from surgical trauma induce immunologic alterations in the patient that depend on the extent of the injury. Laparoscopy, as opposed to open surgery, reduces the systemic inflammatory changes of surgery and this, along with reduced complications, may be the mechanism for preservation of muscle.^22^ The relative preservation of health during the first year after laparoscopic surgery may be the mechanism underlying reports of improved cancer outcomes compared with conventional open colorectal resection. Recent work from our group (Malietzis et al., BJS in print) showed that laparoscopy and myopenia were both independent predictors of survival in CRC patients treated surgically when adjusted for confounding factors such as BMI, age, UICC, and visceral adiposity. This finding, combined with the results of this study, may be extrapolated that increased adoption of laparoscopy for CRC surgery may have a positive indirect impact upon cancer survival.

GCM, the statistical methodology used, is an advanced technique to determine individual growth profiles and to address questions of stability over time, with a number of advantages by comparison with other analytical methods. First, it provides a more flexible way to analyse unbalanced data with measurements that are inconsistent over time; second, it allows investigators to analyse both intra- and intersubject differences in the growth parameters (e.g. slopes and intercepts); third, the effects of predictors at higher levels and other predictors on individual growth can flexibly be added in the GCM; and, finally, the GCM approach is more powerful in examining the effects associated with measures over time as it models the covariance matrix (i.e. fitting the true covariance structure to the data), rather than imposing a certain type of structure as is commonly used in traditional univariate and multivariate approaches.^7^

Limitations of this study include the fact that this was an uncontrolled study despite comprising a relatively large and homogenous data set. Electronic records of CT scans were not available before 2007 as the PACS was introduced in February that year and has contributed to the exclusion from the study of a proportion of the St Mark’s early cohort. BMI and other pathological markers, such as grade of differentiation and lymphovascular invasion, were not included in the analysis as previous work from our group did not identify any significant relationships between these parameters and the presence of muscle depletion in CRC patients.^23^ Finally, the effect of the postoperative outcomes on the LSMI trajectory, and that of additional treatments such as adjuvant chemotherapy, was not interrogated. To avoid confusion with regard to the impact of the postoperative major morbidity events, we analyzed CT scans requested only for CRC follow-up purposes. The time point of the initiation and duration of the adjuvant chemotherapy regimen made it extremely difficult to account for, in an analysis that focused mainly on preoperative factors and their impact on the muscle trajectory.

## Conclusions

In patients undergoing CRC surgery, laparoscopy and the absence of a significantly elevated SIR favors preservation and restoration of muscle mass, postoperatively. The emerging data from our study may permit the development of new treatment protocols whereby monitoring and modifying body composition has therapeutic potential.
